# The effect of preoperative ureteral stenting in retrograde Intrarenal surgery: a multicenter, propensity score-matched study

**DOI:** 10.1186/s12894-020-00715-1

**Published:** 2020-09-14

**Authors:** Hyeong Dong Yuk, Juhyun Park, Sung Yong Cho, Luck Hee Sung, Chang Wook Jeong

**Affiliations:** 1 Department of Urology, Seoul National University Hospital, 101 Daehak - ro, Jongno - gu, Seoul, 03080 Republic of Korea; 2 Department of Urology, Asan Medical Center, University of Ulsan College of Medicine, Seoul, South Korea; 3 Department of Urology, Inje University Sanggye Paik Hospital, 1342 Dongil-ro, Nowon-gu, Seoul, 01757 South Korea

**Keywords:** Preoperative ureteral stenting, Renal stone, RIRS, Ureteral access sheath, Ureteral stent, Urolithiasis

## Abstract

**Background:**

Stent placement before retrograde intrarenal surgery (RIRS) can theoretically expand the ureter to improve access and remove stones. The purpose of this study was to investigate the effect of preoperative ureteral stenting on access and surgery.

**Methods:**

We retrospectively analyzed patients who underwent RIRS between January 2010 and December 2016 at multiple centers. The patients were divided into two groups based on whether or not a ureteral stent was inserted preoperatively. The characteristics of the stone (size, number, density, and location), the success rate of the access sheath placement, perioperative complications, operative times, hospitalization periods, the period for which the stents remained, postoperative urinary tract infection rates, stone-free rates, and additional treatment rates were analyzed.

**Results:**

Overall, 727 patients were included in the study (113 were pre-stented and 614 were non-stented). The median stone size was 12.2 mm. The overall stone-free rate (SFR) was 85.8% for the pre-stented group and 83.2% for the non-stented group, showing no significant (*p* = 0.498) difference between the two groups. Preoperative ureteral stenting improved the success rate of sheath placement (93.8% vs. 85.3%, *p* = 0.023) during surgery. The access sheath size in participants in the pre-stented group showed a tendency to be larger than that in participants in the non-stented group. However, there were no differences in perioperative complications, operative times, additional treatment rates, and stone-free rates.

**Conclusions:**

Although preoperative ureteral stenting did not affect operative outcomes, it increased the success rate of access sheath placement. Depending on the patient’s characteristics, preoperative ureteral stenting can be considered as an adjunctive option when access sheath insertion is considered during RIRS.

## Background

Retrograde intrarenal surgery (RIRS) is currently one of the standard treatments for patients with kidney stones < 2 cm [1]. Advances in technologies such as the development of new flexible ureteroscopes (URS) and small diameter effective lasers have made RIRS an efficient and safe option to manage urinary stones [2]. In stone surgery, a ureteral stent is generally placed after ureteroscopic surgery. It is inserted before ureteroscopic surgery when obstructive uropathy, stone-related complicated infections, or compromised renal function are present [3]. Also, preoperative ureteral stent placement is used when the ureter orifice is too narrow to allow the introduction of the URS.

In the European Association of Urology (EAU) guidelines, the routine placement of ureteral stents prior to RIRS for renal stones is not required. However, several studies have reported that preoperative ureteral stenting affected the outcome of ureteroscopic stone surgery [4–7]. Therefore, we sought to investigate the relationship between preoperative ureteral stent placement and renal stone surgery outcomes.

## Methods

### Study population

The institutional review board at Seoul National University Hospital and SMG-SNU Boramae Medical Center approved this retrospective study and it was exempted from obtaining informed consent. All research and related protocols used in this study complied with the ethical principles of the Declaration of Helsinki.

We retrospectively analyzed the medical records of 727 consecutive patients with renal stones who underwent RIRS between January 2010 and December 2016 at three institutions. Patients with a previous history of RIRS, ureteroscopic lithotripsy, laparoscopic ureterolithotomy, percutaneous nephrolithotomy (PCNL), and pyeloplasty were excluded from the analysis. Patients with ureteral strictures were also excluded.

### Study design

The patients were divided into two groups, the preoperatively stented and the non-stented groups. The preoperatively stented group underwent preoperative ureteral stent placement as follows: The inserted ureteral stent size was routinely 6–7 Fr. The duration of preoperative ureteral stenting was between one and two weeks. All patients underwent RIRS under general anesthesia and prophylactic second-generation cephalosporin antibiotics were generally administered 1 hour before surgery. The flexible URSs used in RIRS were the Olympus URF-P3, URF-V, and Karl Storz Flex-X2 flexible URSs. In all RIRS procedures, a ureteral access sheath (UAS) was inserted prior to flexible URS insertion. The Guidewires used were Cook Medical Roadrunner® PC Hydrophilic Wire Guide, Terumo Guide Wire Radifocus, and Boston Scientific Amplatz Super Stiff™ Guidewire. The UASs used were the Olympus UroPass Access Sheath (10/12-Fr, 12/14-Fr), the Boston Scientific Navigator™ Ureteral Access Sheath (11/13-Fr, 12/14-Fr) 4/16-Fr) and the Cook Medical Flexor® (12/14-Fr, 14/16-Fr).

The most commonly used UAS size was 12/14Fr. If 12/14Fr size was not suitable, then 11/13Fr and 10/12Fr were used. 14/16-Fr UAS was used once in surgery for patients with kidney stones over 2 cm and multiple stones. Lumenis® Pulse™ and VersaPulse® PowerSuite™ 100 W laser systems were used. As for the ureteral stent, a 6Fr double J stent was generally used. Laser fiber sizes were 200 and 365 μm. Laser setting values were long pulse width with 0.4 ~ 2 J and 10 ~ 40 Hz. Postoperative ureter stents were removed one or two weeks postoperatively. The stone-free rates (SFR) were assessed by computed tomography (CT) scans 3 months after surgery. SFR and clinically insignificant residual fragment (CIRF) were defined according to the diameter of the residual stone at three months after surgery (0 mm and <  4 mm, respectively) as seen on CT [8].

For postoperative pain control, paracetamol and tramadol drugs were used. Antibiotics were maintained for up to one week postoperatively, and anticholinergic drug was administered as needed for ureteral catheter related urinary symptom. Additional treatment was defined as the case where another stone surgery or SWL was performed for residual stone within 3 months after RIRS.

For UAS failure cases, first, only flexible URS along the guidewire was inserted. If the flexible URS insertion was done without access sheath, the laser was then inserted and dusting was performed rather than fragmentation. If the UAS was challenging to insert due to the narrow or kinked ureter, reoperation was performed a week after the ureteral stenting. In some cases, balloon catheter dilation was performed when the flexible URS could not enter after ureteral stenting.

### Modified Seoul National University renal stone complexity score (S-ReSC)

In 2011, our institution devised and validated an S-ReSC scoring system to predict the SFR after PCNL [9]. The S-ReSC score is a scoring system that the ratings are calculated by the number of stone involved sites such as the renal pelvis, major calyx, and minor calyx regardless of the size and number of stones. And additional points added for locations where it is difficult to access. Modified S-ReSC was additionally devised and validated to predict SFR after RIRS in 2014. Based on the S-ReSC score, the following groups were obtained: low score group, 1–2 points; medium score group, 3–4 points; and high score group, 5–9 points [10].

### Statistical analyses

The characteristics of the stones (location, size, number, density, laterality and complexity), the success rate of the UAS placement, intraoperative complications, operative times, hospitalization durations, ureteral stenting period, postoperative complications, and SFRs were analyzed. In the statistical analyses, the continuous variables are expressed as the mean value and standard deviations. Categorical variables are expressed as the frequencies of events (%).

Continuous variables are expressed as mean, standard deviation (SD). Baseline characteristics of the two groups were analyzed using Pearson’s Chi-squared test and T-test. Characteristics of renal stones were also analyzed using Pearson’s Chi-squared test and T-test. Factors affecting the SFR were analyzed using univariate and multivariate logistic regression. Propensity score-matching was performed to balance potential confounding variables between the preoperatively stented group and the non-stented group to minimize the selection bias. Nearest neighbor 3:1 propensity score-matching was performed. Propensity scores were calculated by preoperative covariates using multivariate logistic regression analysis of each patient. These covariates were age, BMI, sex, creatinine (Cr), glomerular filtration rate (GFR), hydronephrosis, and characteristics of the stone (complexity, size, number, density, and laterality). Balance between the two groups was assessed by absolute standardized differences before and after matching. All statistical tests were performed using IBM SPSS Statistics, version 22.0 (IBM, Armonk, NY, USA) and a *p*-value < 0.05 indicated statistical significance.

## Results

### Clinical characteristics of the patients according to preoperative ureteral stenting

A total of 727 patients were included in the study, including 614 in the non-stented group (mean age: 55.4 ± 14.2 years) and 113 patients (mean age: 57.5 ± 14.1 years) in the preoperatively stented group. No significant differences were seen between the two groups in age, body mass index (BMI), gender, prevalence of diabetes mellitus (*p* = 0.914), hypertension (*p* = 0.239), cardiovascular disease (*p* = 0.707), or cerebrovascular accidents (*p* = 0.348) and preoperative creatinine and hemoglobin levels. However, estimated glomerular filtration rate (eGFR) and hydronephrosis level were significantly (*p* = 0.002 and < 0.001) lower in the non-stented group than those in the preoperatively stented group. Except for laterality (*p* < 0.001), no significant differences in most stone characteristics, including density, size, and number, were seen between the groups. Modified Seoul National University Renal Stone Complexity scores (S-ReSC) were used to reflect stone complexity. Complexities of the renal stones were significantly higher in patients in the preoperatively stented group than in the non-stented group and the related hydronephrosis was also different between the two groups. (Table 1).

**Table 1:** Baseline characteristics of patients before and after propensity score-matching

	Before propensity score-matching			After propensity score-matching		
Variables	Non-stented group (*n* = 614)	Preoperatively stented group (*n* = 113)	*p*-value	Non-stented group (*n* = 339)	Preoperatively stented group (n = 113)	*p*-value
Mean age (year)	55.4 ± 14.2	57.5 ± 14.1	0.151	57.6 ± 13.0	57.5 ± 14.1	0.949
Mean BMI (Kg/m^2^)	24.8 ± 4.4	24.6 ± 2.9	0.668	24.7 ± 3.3	24.6 ± 2.9	0.842
Gender			0.011			0.594
Male	386 (62.9%)	56 (49.6%)		183 (54.0%)	56 (49.6%)	
Female	228 (37.1%)	57 (50.4%)		156 (46.0%)	57 (50.4%)	
Preoperative Hb	13.5 ± 1.8	13.4 ± 2.0	0.767	13.1 ± 1.8	13.4 ± 2.0	0.208
Preoperative Cr	1.0 ± 0.6	1.1 ± 0.5	0.179	1.1 ± 0.8	1.1 ± 0.5	0.869
Preoperative GFR	79.9 ± 28.0	72.0 ± 24.0	0.002	73.1 ± 22.6	72.0 ± 24.0	0.724
Modified S-ReSC score group			< 0.001			0.464
Low	507(82.6%)	28(24.8%)		117 (34.5%)	28(24.8%)	
Medium	44(7.2%)	13(11.5%)		33 (9.7%)	13(11.5%)	
High	63(10.2%)	72(63.7%)		189 (55.8%)	72(63.7%)	
Laterality			< 0.001			0.088
Right	338 (55.0%)	51 (45.1%)		126 (37.2%)	51 (45.1%)	
Left	276 (45.0%)	62 (54.8%)		213 (62.8%)	62 (54.8%)	
Density of stones (HU)	823.7 ± 352.6	889.3 ± 370.1	0.072	908.3 ± 336.7	889.3 ± 370.1	0.687
Stone max diameter (mm)	12.0 ± 8.5	13.8 ± 9.7	0.058	14.3 ± 9.9	13.8 ± 9.7	0.715
Number of stone			0.232			0.194
1	310 (50.5%)	49 (43.4%)		174 (51.3%)	49 (43.4%)	
2	123 (20.0%)	30 (26.5%)		57 (16.8%)	30 (26.5%)	
≥ 3	181 (29.5%)	34 (30.1%)		108 (31.9%)	34 (30.1%)	
Hydronephrosis	169 (27.5%)	59 (52.2%)	< 0.001	162 (47.8%)	59 (52.2%)	0.425

*BMI* Body mass index, *GFR* Glomerular filtration rate, *S-ReSC* Seoul National University Renal Stone Complexity score, *HU* Hounsfield units, *Hb* hemoglobin, *Cr* Creatinine

### Perioperative outcomes and complications between the preoperatively stented group and non-stented group

The access sheath size in participants in the pre-stented group showed a tendency to be larger than that in participants in the non-stented group. Stone-free rates, CIRF, operative times, and additional treatments were not significantly different between the two groups. However, a significant difference was observed in the access sheath placement success rate among patients who underwent preoperative ureteral stenting. An access sheath was successfully inserted in 524 patients (85.3%) in the non-stented group and in 106 (93.8%) patients in the preoperatively stented group (*p* = 0.023) (Table 2).

**Table 2:** Perioperative outcomes according to preoperative ureteral stenting

	After propensity score-matching		
Variables	Non-stented group (n = 339)	Preoperatively stented group (n = 113)	*p*-value
Operative time (min)	70.2 ± 53.0	65.1 ± 47.4	0.452
Access sheath placement	297 (87.6%)	106 (93.8%)	0.038
Access sheath size (Fr)			0.223
11/13	147 (49.5%)	49 (46.2%)	
12/14	150 (50.5%)	56 (52.8%)	
14/16	0 (0.0%)	1 (0.9%)	
Stone-free rate (0 mm)	282 (83.2%)	97 (85.8%)	0.498
Clinically insignificant residual fragments (< 4 mm)	315 (92.9%)	103 (91.2%)	0.806
Hospitalization period (days)	1.4 ± 1.7	1.3 ± 0.7	0.279
Additional treatment	11 (3.2%)	6 (5.3%)	0.421

Intraoperative and postoperative complications were not associated with preoperative ureteral stenting. Nine grade two or lower complications (8.0%) and two grade three or higher complications (1.8%) occurred in the preoperatively stented group. Thirty-three grade two or lower complications (9.7%) and one grade three or higher complications (0.4%) occurred in the non-stented group (Table 3). Three (2.7%) intraoperative complications occurred in the preoperatively stented group, intrarenal bleeding one (0.9%), ureteral perforation one (0.9%), and arrhythmia one (0.9%). Forty-seven (7.6%) postoperative complication occurred patients in the non-stented group and eight (7.1%) patients in the preoperatively stented group.

**Table 3:** Perioperative complications according to modified Clavien classification system

	After propensity score-matching		
Variables	Non-stented group (n = 339)	Preoperatively stented group (n = 113)	*p*-value
Intraoperative complication			
Grade I			
Intrarenal bleeding	9 (2.7%)	1 (0.9%)	0.410
Grade II			
Arrhythmia	0 (0.0%)	1 (0.9%)	0.845
Grade III			
Ureter perforation	0 (0.0%)	1 (0.9%)	0.872
Postoperative complication			
Grade I			
Fever	9 (2.7%)	2 (1.8%)	0.664
Grade II			
Blood transfusion	0 (0.0%)	0 (0.0%)	NA
Urinary tract infection	9 (2.7%)	5 (4.4%)	0.519
Acute pyelonephritis	6 (1.8%)	0 (0.0%)	0.478
Grade III			
Ureteral stricture	1 (0.3%)	1 (0.9%)	0.965
Grade IV			
Sepsis	0 (0.0%)	0 (0.0%)	NA

The rate of postoperative infections was similar in both groups. A postoperative UTI and acute pyelonephritis occurred in 25 (4.1%) patients in the non-stented group and five (4.4%) in the preoperatively stented group. All 30 postoperative UTI and acute pyelonephritis cases were revisited after discharge and were treated with antibiotics after confirmation with a positive urine test. There was one postoperative sepsis in the non-stented group (Table 3).

### Clinical characteristics of the patients and perioperative outcomes and complications in propensity score-matching

In propensity score-matching between the two groups, no significant differences were seen in patient and stone characteristics between the two groups (Table 1). And perioperative outcomes, such as stone-free rates, clinical stone-free rates, operative times, and hospitalization periods were not significantly different between the two groups. Intraoperative and postoperative complications were not significantly different between the two groups (Table 2). However, the access sheath placement success rate was higher in patients who underwent preoperative ureteral stenting (Table 2).

### Multivariate logistic regression analyses for factors affecting the stone-free rate

The multivariate regression analyses of factors affecting the SFR showed that stone characteristics, such as size and density of the stone and modified S-ReSC scores were significantly related to stone-free rates (Table 4). Access sheath placement was also significantly related to stone-free rates. In addition to stone size, density, complexity, and access sheath placement were also significant predictors of SFRs after propensity score-matching (Table 4).

**Table 4:** Multivariate logistic regression of stone-free rate predictors

Variables	After propensity score-matching	
	OR (95%Cl)	*p*-value
BMI	0.98 (093–1.04)	0.570
Preoperative DJ stenting	1.07 (0.49–2.32)	0.861
Laterality	0.91 (0.43–1.93)	0.805
Number of stones	0.92 (0.78–1.09)	0.348
Stone max diameter	0.95 (0.90–0.99)	0.028
Density of stones	0.99 (0.99–1.00)	0.007
Modified S-ReSC score	0.42 (0.29–0.75)	0.002
Hydronephrosis	0.91 (0.46–1.79)	0.781
Access sheath placement	1.27 (1.08–1.88)	0.029

*BMI* Body mass index, *S-ReSC* Seoul National University Renal Stone Complexity score

## Discussion

In 1987, Bagley first introduced RIRS and reported the results of a flexible RIRS procedure [11]. The development of optical technology, surgical methods, and instruments for RIRS have improved to the point where the procedure is now considered the primary treatment option for patients with kidney stones less than 2 cm [1, 7, 12, 13]. Among the advances that have been made, the access sheath has played a significant role in RIRS. The access sheath allows the flexible URS to quickly and repeatedly enter the kidney and upper ureter and also reduces the risk of injury to the ureter. It also prevents pyelovenous reflux of large amounts of perfusion during surgery [14–18]. The access sheath has been shown to reduce intrarenal pressure and improve vision [14, 19]. Although the usefulness of the access sheath is well known, it is not available for all surgeries. Mogilevkin et al. reported that the failure rate for access sheath placement was approximately 15% [20]. That study only evaluated cases in which a 14Fr access sheath was inserted and preoperative ureteral stenting was effective. In our study, we observed a 93.8% success rate for access sheath placement in the preoperatively stented group and 85.3% in the non-stented group (*p* = 0.023). These findings suggest that preoperative ureteral stenting increased the success rate of access sheath placement. Preoperative ureteral stenting is thought to cause passive ureter dilation. A dilated ureter increases the probability of access sheath placement. Several studies have reported that preoperative ureteral stenting affected the outcomes of patients who underwent RIRS [21]. Purlmutter et al. reported that preoperative stents dilated the ureter, passively affecting the outcomes of RIRS [22], while Rubenstein et al. reported that there was a significant effect on the stent and SFR [23]. However, Fabrizio et al. reported that preoperative ureteral stenting affected the expansion of the ureter but there was no significant correlation with stone clearance [24].

In our study, the SFR was 82.7% in the non-stented group and 85.8% in the preoperatively stented group (*p* = 0.379). The CIRF rate was 93.0% in the non-stented group and 91.2% in the preoperatively stented group (*p* = 0.619). In propensity score-matching, the SFR was 83.2% in the non-stented group and 85.8% in the preoperatively stented group (*p* = 0.498). The CIRF rate was 92.9% in the non-stented group and 91.2% in the preoperatively stented group (*p* = 0.806). These results indicate that preoperative ureteral stenting was not significantly associated with stone clearance. In multivariate logistic regression, stone characteristics such as size, density, and complexity affected the stone-free rate. In addition to stone characteristics, access sheath placement has been shown to affect the stone-free rate. Propensity score- matching results also showed that stone size, density, complexity, and access sheath placement were important predictors of SFRs (Table 4). However, preoperative ureteral stenting had no significant effect on SFRs. Most RIRS surgeries are performed without preoperative ureteral stenting. In general, preoperative ureteral stenting is considered when UAS insertion is difficult or when it is difficult to insert flexible URS directly. Although ureteral preoperative stenting did not affect SFR, it increased the success rate of UAS insertion. This might be an important source for the prediction of patients who need preoperative stenting in the future. Large-scale prospective studies related to the prediction of patients who need preoperative stents need to be performed in the future.

In our study, patients in the preoperatively stented group had fewer overall complications than the non-stented group, although the difference between the two groups was not statistically significant (Grade I-II complication rates: 33 (9.7%) versus 9 (8.0%); grade III-IV complication rates: 0.3% versus 1.8%) (Table 3). One patient (0.9%) developed ureter perforation who was a preoperatively stented patient. In a previous study, patients in the pre-stented groups had fewer complications than those in the non-stented group (major complications: 0.6% vs. 1.6%; minor complications: 4.7% vs. 9.4%) [4]. Ureter perforation was also relatively lower in the pre-stented group (2.7% vs. 9.4%) [4]. Similar to our results, Rubenstein et al. have also shown no significant difference in the rate of complications between the two groups [23]. Lee et al. have compared a short preop-stenting group, a long preop-group, and a no-stenting group and found no significant difference in overall complication among the three groups [25].

We acknowledge that our study had limitations. First, we used a retrospective study design. Second, this study was not free from selection bias due to the use of multiple laser systems and laser fibers, multiple types and sizes of ureteric access sheathes, multiple types of URS, and multiple surgeons. Third, we did not insert the preoperative stents randomly, therefore, a selection bias could have occurred. Patients in the preoperatively stented group had more complex stone positions of than those in the non-stented group and the degree of hydronephrosis was higher in this group. Fourth, UAS is not a routine practice in flexible URS. Some endourologists prefer to go directly with a flexible URS over a guidewire. Therefore, propensity score-matching was performed for calibration. If these limitations are addressed in future studies, the results are expected to be more significant.

## Conclusions

This study showed that preoperative ureteral stenting increased the success rate of access sheath placement. However, preoperative stenting had no significant effect on operative outcomes or complications such as SFRs, operative times, perioperative complications, or ureteral strictures.

## Data Availability

The datasets used and/or analysed during the current study are available from the corresponding author upon reasonable request.

## Abbreviations

BMI: Body mass index
CT: Computed tomography
eGFR: Estimated glomerular filtration rate
HU: Hounsfield units
RIRS: Retrograde intrarenal surgery
S-ReSC: Seoul National University Renal Stone Complexity score
UTI: Urinary tract infection
